# Renal Denervation after Symplicity HTN-3: An Update

**DOI:** 10.1007/s11906-014-0460-x

**Published:** 2014-06-10

**Authors:** Alexandre Persu, Yu Jin, Fadl Elmula Mohamed Fadl Elmula, Lotte Jacobs, Jean Renkin, Sverre Kjeldsen

**Affiliations:** 1 Pole of Cardiovascular Research, Institut de Recherche Expérimentale et Clinique, Université Catholique de Louvain, Brussels, Belgium; 2 Division of Cardiology, Cliniques Universitaires Saint-Luc, Université Catholique de Louvain, 10 Avenue Hippocrate, 1200 Brussels, Belgium; 3 Studies Coordinating Centre, Research Unit Hypertension and Cardiovascular Epidemiology, KU Leuven Department of Cardiovascular Sciences, University of Leuven, Leuven, Belgium; 4 Department of Cardiology, Ullevål University Hospital, University of Oslo, Oslo, Norway

**Keywords:** Renal denervation, Resistant hypertension, Ambulatory blood pressure, White coat effect, Blinding, Renal artery stenosis

## Abstract

After three years of excessive confidence, overoptimistic expectations and performance of 15 to 20,000 renal denervation procedures in Europe, the failure of a single well-designed US trial—Symplicity HTN-3—to meet its primary efficacy endpoint has cast doubt on renal denervation as a whole. The use of a sound methodology, including randomisation and blinded endpoint assessment was enough to see the typical 25–30 mmHg systolic blood pressure decrease observed after renal denervation melt down to less than 3 mmHg, the rest being likely explained by Hawthorne and placebo effects, attenuation of white coat effect, regression to the mean and other physician and patient-related biases. The modest blood pressure benefit directly assignable to renal denervation should be balanced with unresolved safety issues, such as potentially increased risk of renal artery stenosis after the procedure (more than ten cases reported up to now, most of them in 2014), unclear long-term impact on renal function and lack of morbidity–mortality data. Accordingly, there is no doubt that renal denervation is not ready for clinical use. Still, renal denervation is supported by a strong rationale and is occasionally followed by major blood pressure responses in at-risk patients who may otherwise have remained uncontrolled. Upcoming research programmes should focus on identification of those few patients with truly resistant hypertension who may derive a substantial benefit from the technique, within the context of well-designed randomised trials and independent registries. While electrical stimulation of baroreceptors and other interventional treatments of hypertension are already “knocking at the door”, the premature and uncontrolled dissemination of renal denervation should remain an example of what should not be done, and trigger radical changes in evaluation processes of new devices by national and European health authorities.

## Introduction

In 2009, Krum and colleagues [1] published a non-randomised proof-of-concept study, testing percutaneous radiofrequency catheter-based renal sympathetic denervation (RDN) as a novel treatment of resistant hypertension in a cohort of 45 patients. RDN proved feasible, effective (blood pressure decrease: -27/17 mmHg at 12 months) and safe. One year later, the Symplicity HTN-2 investigators confirmed these impressive results in an open-label randomised trial including 106 patients with resistant hypertension. One hundred patients were assessed for the primary endpoint at 6 months: in the RDN group (*n* = 49), office blood pressure decreased by 32/12 mmHg (*P* < 0.0001), whereas blood pressure (BP) remained unchanged in the control group (*n* = 51) (+1/0 mmHg, *P* ≥ 0.77). Accordingly, at 6 months the between-group difference in office blood pressure averaged 33/11 mmHg (*P* < 0.0001) [2••]. There were no serious procedure-related or device-related complications and occurrence of adverse events did not differ between groups [2••]. Similar blood pressure decreases were documented 6 months after RDN, both in the Symplicity HTN-1 registry [3] (-25/11 mmHg) and a meta-analysis including mostly uncontrolled studies (-25/10 mmHg) [4].

Still, in this sunny landscape, many shadows remained. Only a minority of patients with resistant hypertension were found to be eligible for RDN [5, 6, 7•]. Blood pressure response to RDN was highly variable [8]. Besides baseline blood pressure, no predictors of response to RDN were consistently found. In particular, clinical situations associated with sympathetic overactivity such as diabetes, metabolic syndrome or chronic kidney diseases were not associated with an increased likelihood of blood pressure response to RDN [2••, 3, 8]. On the contrary: in the European Network COordinating research on Renal Denervation (ENCOReD) registry, baseline plasma creatinine was inversely correlated with blood pressure decrease 6 months after RDN [8]. After exclusion of non-adherent patients, the residual benefit of RDN was minimal [9]. Ambulatory blood pressure decrease after RDN was seldom reported, and when reported was not always significant [10, 11]. In the largest cohorts where both office and out-of-the office blood pressure were measured, ambulatory blood pressure decrease was only one-third of office blood pressure decrease [8, 12], versus an expected 60–70 % in drug trials [13, 14]. Finally, the lack of any blood pressure decrease in the control group of Symplicity HTN-2 [2••] was a matter of concern [15]. As a comparison, in three contemporary randomised studies performed in patients with resistant hypertension [16–18], office systolic blood pressure in the control group decreased by 8, 9 and 14 mmHg, respectively.

As early as 2012, we and others [13, 15] have stressed some of these discrepancies, suggesting that part of the apparent benefits of RDN may reflect non-specific effects such as placebo effect, Hawthorne effect, decreasing white-coat effect, regression to the mean and other patient or physician-related biases. It was also suggested that the differential outcome between RDN and placebo arms would substantially shrink if assessment of efficacy was blinded, either using 24-h ambulatory blood pressure as primary endpoint [8, 13], or a sham procedure [19••].

## Renal Denervation before Symplicity HTN-3

Despite these caveats, publication of the Symplicity studies [1, 2••, 3] was followed by an unprecedented wave of enthusiasm. Medtronic Inc® (Minneapolis, Minnesota) paid $800 million upfront to purchase Ardian® (Mountain View, California), the company that had developed the technology [20]. More than ten companies developed their own RDN system, five of which obtained the Conformité Européenne (CE) mark. The procedure was quickly reimbursed in Germany, and later on in Switzerland, Sweden and the Netherlands. While RDN remained an investigational procedure in the US, 15 to 20,000 procedures were performed in Europe in less than 4 years [21••], most of them using the Ardian-Medtronic® catheter. As icing on the cake, RDN was recommended by the European Society of Hypertension (ESH) with a grade IIC recommendation for treating patients with resistant hypertension [22].

Based on mostly small, purely observational studies, RDN was associated with ancillary benefits such as decreased left ventricular mass [23], arterial stiffness [24], heart rate and atrioventricular conduction [25, 26], arrhythmia burden [27], renal resistive indices [28] and albuminuria [29], as well as with improved cardiac function [23], central hemodynamics [24] and insulin sensitivity [30]. Furthermore, it was proposed as a potential treatment in a host of medical conditions, including chronic kidney disease [31], atrial fibrillation [32], heart failure [33], obstructive sleep apnoea [34] and polycystic ovary syndrome [35]. Finally, in spite of cautionary recommendations by European [36] and international [37] expert panels, RDN was applied to patients with white-coat resistant hypertension [12], isolated systolic hypertension [38], stenotic [39] or stented [40] renal arteries and even—inadvertently—in a patient with Münchausen syndrome [41]. New papers devoted to RDN were published weekly, if not daily, and all top-ranking journals in the field were keen to host studies, reviews and editorial comments addressing this novel approach [20]. In the meantime, dissenting and cautionary advice [13, 15] and predictions [19••] remained almost unheard in the general chorus of praise.

## The Earthquake of Symplicity HTN-3

Symplicity HTN-3 [42••], a large US randomised controlled trial including 535 patients assigned in a 2:1 ratio to RDN or a sham procedure, was to be the last step of the triumphal march of RDN. Accordingly, the announcement that Symplicity HTN-3 failed to meet its primary efficacy endpoint (http://www.tctmd.com/show.aspx?id=123265) stunned the medical community and device industry as a deep shock, and was quickly followed by definitive or temporary stopping of large ongoing research programmes (http://www.reuters.com/article/2014/01/24/us-covidien-divestment-idUSBREA0N18B20140124). A rapid opinion shift from excessive optimism to deep mistrust ensued [43].

**Fig. 1:**
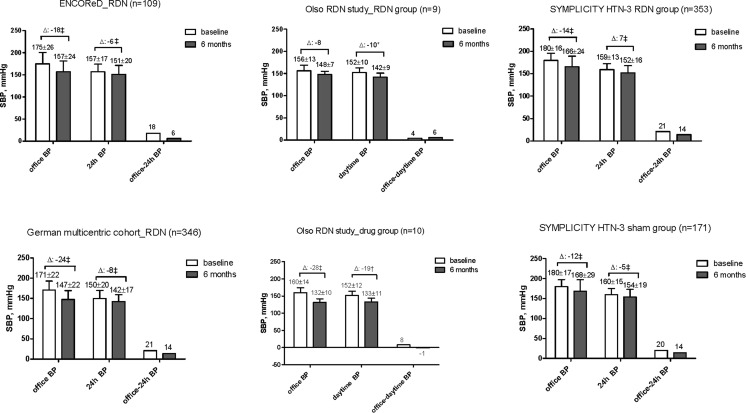
The figure compares changes in office blood pressure, ambulatory blood pressure and white coat effect (office blood pressure–ambulatory blood pressure) between baseline and 6 months in patients who underwent renal denervation in the European RDN cohort [8], the multicentre German RDN cohort [12] and the Oslo RDN [21] and Symplicity HTN-3 [42] randomised control trials. It highlights the following points: (1) with the exception of office blood pressure in the Oslo-RDN trial, baseline blood pressures are very similar; (2) while office blood pressure changes largely vary from one study to the other, ambulatory blood pressure decrease is highly consistent, between 6 and 10 mmHg; (3) in agreement with our previous analysis comparing blood pressure decrease in the ENCOReD RDN cohort and the placebo and treatment arms of the Syst-Eur trial [8], blood pressure decrease after RDN seems to be intermediary between decreases observed in placebo and drug treatment adjustment groups. However, formal demonstration would require a three-arm randomised trial comparing directly RDN, drug treatment adjustment and continuation of baseline drug treatment; (4) with the exception of the Oslo RDN trial, where baseline difference between office and ambulatory blood pressure was < 5 mmHg, white coat effect typically decreased by 18–21 mmHg 6 months after RDN, which represents twice the 10 mmHg decrease proposed by the Symplicity investigators to define blood pressure response to RDN; (5) the attenuation of white coat effect at six months was similar in the RDN and placebo arms of Symplicity HTN-3, and larger in the drug adjustment than in RDN arm of the Oslo RDN trial. These observations confirm that the large discrepancy between office and ambulatory blood pressure changes observed in most RDN trials reflects non-specific effects [8] rather than an effect of RDN per se, as hypothesized by Doumas et al. [14]. BP: blood pressure; ENCOReD: European Network COordinating research on Renal Denervation; RDN: renal denervation; SBP: systolic blood pressure

In contrast with Symplicity HTN-2 [2••], in Symplicity HTN-3 [42••], the mean decrease in office systolic blood pressure at 6 months was only 14.13 mmHg in the RDN group, as compared with a substantial 11.74 mmHg decrease in the sham group (*P* < 0.001 for both comparisons of the change from baseline). This corresponds to a modest 2.39 mmHg advantage in favour of the technique, lower than the preset superiority margin of 5 mmHg [42••]. Discordant results were also observed for ambulatory blood pressure, particularly in the control group. While in Symplicity HTN-2, 24-h ambulatory systolic blood pressure decrease was -11 mmHg in 20 patients treated with RDN (*p* = 0.006) compared to a non-significant (*p* = 0.51) -3 mmHg in 25 patients from control group [2••], in Symplicity HTN-3, 24-h ambulatory blood pressure decrease was modest but significant and of the same order of magnitude in both groups (-6.75 mmHg and -4.79 mmHg, respectively; *p* < 0.001 for both) [42••] (Fig. 1).

The huge discrepancy between Symplicity HTN-3 on one side and most other studies including Symplicity HTN-2 on the other side raises concern. Can a single large US trial challenge all previous studies? Who is right? Symplicity HTN-2, Symplicity HTN-3, or both? Compared to Symplicity HTN-2 [2••], patients included in Symplicity HTN-3 [42••] were more overweight (body mass index: 34 vs. 31 kg/m^2^), at higher cardiovascular risk (slightly higher proportion of type 2 diabetes and dyslipidemia) and took more diuretics (99 vs. 89 %) and aldosterone antagonists (23 vs. 17 %). Furthermore, as expected in a US population, the Symplicity HTN-3 cohort included one-fourth of patients of African descent [42••], while this proportion was negligible in Symplicity HTN-2 [2••]. The catheter used in Symplicity HTN-3 [42••] was the second generation Medtronic Symplicity catheter rather than the original, stiffer Ardian catheter used in Symplicity HTN-2 [2••]. Finally, as most Symplicity HTN-3 investigators performed their first RDN within the trial, they might have been less experienced that the Symplicity HTN-1 and 2 investigators [42••].

The aforementioned differences in study populations and in the RDN procedure itself may partly account for the less important blood pressure decrease in RDN-treated patients from Symplicity HTN-3 [42••] compared to previous studies. However, they cannot explain the most striking difference between Symplicity HTN-2 [2••] and Symplicity HTN-3 [42••]: while in Symplicity HTN-2, blood pressure remained unchanged in the placebo arm, in Symplicity HTN-3 it decreased to almost the same extent as in the RDN arm. The single credible explanation is that Symplicity HTN-3 was *blinded*, while other studies were randomised but unblinded (Symplicity HTN-2) or purely observational [19••, 42••]. By contrast with Symplicity HTN-3, open-label studies such as Symplicity HTN-2 are subject to expectation, performance and evaluation biases [15]. In other words, knowing to which treatment group participants are allocated may have affected both physicians’ and patients’ behaviour, particularly if they felt that RDN was the last option available, as implied by the concept of resistant hypertension, if they strongly believed in the efficacy of the technique, or were newcomers in the field of hypertension and blood pressure measurement. Symplicity HTN-2 investigators may have been inclined to measure blood pressure differently in the two groups: for example, the resting period before office BP measurements and the number of BP measurements taken may not have been the same in the RDN and control arms [15]. As detailed elsewhere [13], the Symplicity HTN-2 protocol for blood pressure measurement and recording made this trial particularly vulnerable to such kind of biases. On the other side, being in the RDN group, and as such benefitting from increased attention from the caring physician, may have improved adherence to drug treatment [13, 15, 19••]. Conversely, patients from the control arm may have been tempted not to take properly their medications in order to benefit from RDN after assessment of the primary endpoint at 6 months [13, 15]. In contrast, in a blinded study such as Symplicity HTN-3 [42••], such biases are minimised. In particular, improved adherence to drug treatment is likely to have occurred irrespective of the treatment arm, accounting for part of the substantial blood pressure decrease in the control group [44].

Some of the most enthusiastic exponents of RDN are already ready to “seek redemption” for RDN [45], without fully accepting the lessons of Symplicity HTN-3. While it was previously assumed that RDN was rather easy to perform, they put forward the lack of experience of Symplicity HTN-3 investigators [42••]. They suggest that patients in Symplicity HTN-3 were not the right ones [46] (which is likely, but also applies for previous studies) [47], that selection based—even partly—on ambulatory blood pressure may be inappropriate [46], and that, after all, if RDN is less effective than expected, it may have benefits beyond blood pressure [48] and cure other diseases. However, they forget to tell that these purported benefits are based on observational studies, similar to those that helped to build up the premature success of RDN as an antihypertensive treatment [49]. As for blood pressure, demonstration of benefits of RDN beyond blood pressure can be achieved only within the context of well-designed randomised controlled studies [13]. Notably, while three studies—two based on cardiac echography [23, 48] and one on magnetic resonance imaging (MRI) [50]—suggest a beneficial effect of RDN on left ventricular structure and cardiac function, 3 years after publication of Symplicity HTN-2, the results of the cardiac MRI substudy at 6 months are still not in the public domain.

The key message of Symplicity HTN-3 [42••] is simple and we should be wise enough to accept it: in patients meeting the entry criteria of the study (and probably those of Symplicity HTN-1 and 2 as well), the true overall benefit of RDN on systolic blood pressure is modest, < 3 mmHg, without evidence of a favourable impact on morbidity–mortality thus far. All the rest is common to the placebo and RDN groups, and thus represents “noise”, whatever it means. These findings are reinforced by those of the recently published Oslo RDN trial [21••], stopped early in view of the dramatic superiority of adjusted drug treatment vs. RDN. In view of these results, enthusiastic pharmaco-economic [51, 52] and quality-of-life [53] analyses have collapsed like a house of cards because they are based on weak assumptions. RDN is not ready for clinical deployment and reimbursement is at least questionable.

On the other side, reason takes back her rights. All previous discrepancies are now easily understandable. If the blood pressure decrease observed in previous studies is mostly related to patient and physician-related biases, it is not unexpected that blood pressure outcome may critically depend on the expertise of centres, the way in which blood pressure is measured, and/ or the individual characteristics and psychology of patients. It is obvious that ambulatory blood pressure, which minimises observer-related bias and placebo effect [54], will be much less affected than office blood pressure. Finally, it is not surprising that, besides baseline blood pressure, few consistent predictors of response have been identified [2••, 3, 8]. This is particularly true for Symplicity studies and registry [2••, 3]. Indeed, in the latter, blood pressure response to RDN was defined as a decrease in office systolic blood pressure of ≥ 10 mmHg, which is less than the decrease in white coat effect at 6 months in the ENCOReD [8] and German multicentre [12] cohorts (18 and 21 mmHg, respectively), and inferior to the blood pressure decrease in the sham group of Symplicity HTN-3 [42••] (-14 mmHg) (Fig. 1).

## A Closer Look at Safety Issues

The first announcement of the failure of Symplicity HTN-3 [42••] to achieve its primary endpoint was accompanied by a second statement on the fact that the primary safety endpoint was met (http://www.tctmd.com/show.aspx?id=123265). At first sight, this may convey the impression that the Simplicity HTN-3 investigators have achieved at least one of their aims after all, and thus mitigate our disappointment. However, it should be pointed out that the primary safety endpoint—a composite of major adverse events, including death, endstage renal disease, an embolic event resulting in end-organ damage, renal-artery or other vascular complications, hypertensive crisis within 30 days or new renal-artery stenosis of more than 70 % within 6 months [42••]—was an easy target to reach. Indeed, an increased incidence of death or major cardiovascular events after RDN is unlikely and would have been already detected in existing registries. It was also unlikely that RDN would be associated with more hypertensive crisis than in the sham group. Finally, as in previous Symplicity studies [1, 2••, 3, 55], in the absence of systematic computed tomography (CT) or MRI, a substantial proportion of renal artery stenosis may have been overlooked [13].

Up to now, at least 13 cases of significant, de novo renal artery stenosis have been documented 3 to 6 months after RDN performed using four different renal ablation systems [2••, 55–65]. The diagnosis was made following blood pressure increase after an initial decrease [2••, 56–65], renal function degradation [59, 61] and/or repeated pulmonary oedema [61, 63]. Notably, most of these reports were published in 2014, i.e., after the Medtronic announcement that Symplicity HTN-3 had failed to reach its primary endpoint. Systematic CT angiography after RDN was performed in only three small cohorts, with a prevalence of renal artery stenosis of 2/11 (18 %) [64]; 3/46 (6.6 %) [61, 66]; and 2/15 (13.3 %) (http://www.tctmd.com/show.aspx?id=120456), respectively. If confirmed on a larger scale, this frequency is not negligible, especially in view of the modest efficacy of the technique [21••, 42••]. It may prove even higher if one includes cases of progression of non-significant baseline stenosis [55, 67], some of which could have been favoured by RDN.

Long-term evolution of renal function is also a matter of concern. While glomerular filtration rate (GFR) at 6 months was similar in both RDN and control groups in Symplicity HTN-2 [2••] and Symplicity HTN-3 [42], in the Symplicity HTN-1 registry [55], eGFR loss at 36 months was estimated to be 9.3 ml/min/1.73 m^2^, a twofold to eightfold larger decrease than in other contemporary trials including patients at high cardiovascular risk (median follow-up: 29 to 56 months) [68–70]. Whether this evolution reflects the natural history of renal function in patients with severe, resistant hypertension, increased use of diuretics and/or renin angiotensin inhibitors or a deleterious effect of RDN per se remain to be determined.

## Conclusion

### Take-Home Messages for Clinical Practice

In patients with resistant hypertension as a whole, the efficacy of RDN has been largely overestimated [42••]. Overall, it is clearly inferior to expert drug treatment adjustment [21••]. While a minority of patients may show an impressive response to RDN [8], predictors of response are largely unknown [2••, 3, 8]. Accordingly, it is obvious that RDN is not ready for clinical use. Is this news really bad news? Not really. The prevalence of apparently resistant hypertension—i.e., uncontrolled hypertension despite intake of three antihypertensive drug classes—is estimated to be between 3 and 30 % of the hypertensive population [71–73], with figures of less than 10 % probably representing the true prevalence [22]. It may shrink dramatically (below 5 %) if the definition is restricted to those subjects whose blood pressure remains uncontrolled despite the use of a triple therapy at maximal dosage, including a diuretic [73], and even more when patients with secondary or white coat resistant hypertension (30–40 %) [72] and poorly adherent patients [74••, 75] are excluded. Within the remaining subgroup of “truly resistant” hypertensive patients, a substantial proportion is still amenable to blood pressure control in expert centres [7•].

The proportion of patients in whom RDN appears as the single option is thus very low (probably < 1 %) [5, 73]. Furthermore, many of them will not qualify for the procedure due to anatomical reasons [76], or are unlikely to benefit from RDN because of advanced renal failure [8] or increased arterial stiffness associated with older age.

### Take-Home Messages for Research

While, as stated in a recent editorial, the “RDN bubble” has exploded [44] and overoptimistic views have been abandoned, this does and should not mean the end of RDN [43]. Most patients with resistant hypertension are indeed amenable to blood pressure control after skillful treatment adjustment. However, complex drug treatments are not always well tolerated in the long run. Drug adherence is inversely correlated with the number of drugs [77] and steadily decreases over time [78]. Furthermore, complex therapies imply frequent blood samples and medical visits, and are thus costly and time consuming. On the other side, RDN rests on a solid *rationale* [79]. Most investigators have witnessed at least a few cases of impressive blood pressure decrease after RDN [8]. Hence, there is still an unmet need in a minority of hypertensive patients. RDN is not the miracle cure that was promised [13], but may prove a solution in at least some of them. The challenge will be to identify those few high-risk patients who may benefit from RDN. Notably, in Symplicity HTN-3 [42••], while the distribution of blood pressure response has not been reported so far, analysis of pre-specified subgroups suggests that RDN may be superior to drug treatment alone in several instances, including age < 65 years old and eGFR > 60 ml/min/1.73 m^2^ [42••]. These findings are consistent with analysis of the ENCOReD multicentre cohort, showing an inverse correlation between baseline plasma creatinine and blood pressure decrease after RDN [8].

Identification of determinants of response to RDN should be done within the context of properly designed randomised controlled trials and independent registries. Selection criteria should be much stricter than previously. Candidates to RDN should undergo an in-depth screening in order to rule out secondary and white coat hypertension. Elderly patients, patients with isolated systolic hypertension and altered renal function [8] are unlikely to respond, and should probably be excluded. Drug adherence should be assessed throughout the trial, using electronic pill monitoring, or preferably, drug dosages in plasma or urine. Completeness of RDN should be assessed using methods such as heart rate variability or electrical stimulation of renal arteries [80•]. The primary efficacy endpoint should be based on ambulatory, not office, blood pressure. Indeed, ambulatory blood pressure is blinded by definition, and accordingly minimises white coat and placebo effects and physician-related biases. [54]. Furthermore, ambulatory blood pressure is an independent predictor of cardiovascular events, while after adjustment for conventional cardiovascular risk factors, office blood pressure has little added value [54, 81•]. Office blood pressure measurement in patients blinded for treatment arm as in Symplicity HTN-3 [42••] is an alternative, but is much more complex, exposes patients from the control group to an unnecessary invasive procedure, and has a lesser predictive value than out-of-the office blood pressure measurements. Follow-up should be extended to several years, and primary endpoint for safety should be based on eGFR. The incidence of renal artery stenosis or stenosis progression should be evaluated by CT scan, which is the gold standard [82], or if contra-indicated, by MRI. In order to meet these requirements, we have designed the INSPiRED study [47]. Along with other ongoing trials such as REDUCE-HTN (NCT01570777), INSPiRED is expected to provide new, unbiased information on the efficacy and safety of RDN. Additional information may be derived from comparison of baseline characteristics of extreme-blood pressure responders versus poor or non-responders to the technique, as defined according to ambulatory blood pressure changes after RDN. This approach is currently being tested with the ENCOReD cohort [83].

### General Lessons

RDN may find its place in the therapeutic arsenal of resistant hypertension and some other diseases characterised by sympathetic overactivity. It will never be a “miracle cure” of hypertension, or even the standard treatment for all patients with resistant hypertension [13, 44]. In this sense, and in this sense only, RDN has not met its expectations. The RDN “story” will remain a textbook case for methodologists, epidemiologists and sociologists interested in the process of medical discovery and dissemination of knowledge. They will try to understand how, in the 21st century, shaky evidence derived from a few small trials with suboptimal design convinced a large majority of physicians, scientists and policy makers that RDN was ready for large scale clinical use, until announcement of the failure of a single large trial induced a radical and immediate opinion shift to an equally unmotivated radical mistrust [43].

Different factors have jointly contributed to the uncontrolled and premature deployment of RDN in Europe: (1) the attractive underlying *rationale* [79]; (2) the strong support of top-ranking experts of sympathetic system; (3) the enthusiasm of interventional cardiologists and radiologists looking for new areas of development in an era of “penury”; (4) the unrelenting pressure of industrials seeking new markets [84]; (5) the suboptimal management of resistant hypertension, both in Europe [7•] and the US [85]; (6) the lack of new drugs in the field of hypertension and the disinvestment of most drug companies from the hypertension field; (7) the “magic” effect of the word “resistant hypertension”, which conveys the impression that affected patients are hopelessly exposed to a major risk of potentially fatal cardiovascular complications in the absence of new therapeutic options [13, 86]; and (8) the lack of strict regulations for introduction of device therapies in Europe [13].

Thriving on what is viewed as the failure of RDN to meet its expectations, new treatment approaches of resistant hypertension such as electrical stimulation of baroreceptors [87] or creation of arterio-venous fistulas [88] are “knocking at the door”. In order to avoid a rehearsal of the RDN story, several lessons need to be learned: (1) the most promising hypothesis, with the most solid underlying rationale should be tested using a rigorous methodology [44]; (2) hypertension specialists should be more united in order to deliver authoritative and independent advice and make the difference with industrials, policy makers and interventionalists; (3) the definition of resistant hypertension should be more stringent and primarily based on ambulatory blood pressure [13, 81•, 86]; (4) methodological guidelines for state-of-the art evaluation of new drugs or device treatments of resistant hypertension should be proposed; (5) continued education programmes should help to improve management of resistant hypertension; and (6) new, more strict regulations for the introduction of medical devices in Europe are urgently needed.
